# An interview study about how nurses and physicians talk about the same concepts differently

**DOI:** 10.1186/s12909-024-05682-x

**Published:** 2024-06-26

**Authors:** Ragnhild Holgaard, Birgitte Bruun, Frederik Zingenberg, Peter Dieckmann

**Affiliations:** 1 Center for Human Resources, Copenhagen Academy for Medical Education and Simulation (CAMES), Capital Region of Denmark, Herlev Hospital, 25th floor, Herlev Ringvej 75, Herlev, 2370 Denmark; 2 Department of Public Health, Copenhagen University, Øster Farimagsgade 5, Copenhagen, 1353 Denmark; 3 Department of Quality and Health Technology, University in Stavanger, Kjell Arholms Gate 43, Stavanger, 4021 Norway

**Keywords:** Concept formation, Decision-making, Education of healthcare professionals, Faculty development, Leadership, Social and cognitive capabilities

## Abstract

**Background:**

How healthcare professionals understand and use concepts of social and cognitive capabilities will influence their behaviour and their understanding of others’ behaviour. Differing understandings of concepts might lead to healthcare professionals not acting in accordance with other healthcare professionals’ expectations. Therefore, part of the problem concerning errors and adverse incidents concerning social and cognitive capabilities might be due to varying understandings of concepts among different healthcare professionals. This study aimed to examine the variations in how educators at the Copenhagen Academy for Medical Education and Simulation talk about social and cognitive capabilities.

**Methods:**

The study was conducted using semi-structured interviews and directed content analysis. The codes for the analysis process were derived from existing non-technical skills models and used to show variations in how the participants talk about the same concepts.

**Results:**

Educators with a background as nurses and physicians, talked differently about *leadership* and *decision-making*, with the nurses paying greater attention to group dynamics and external factors when describing both *leadership* and *decision-making*, whereas physicians focus on their individual efforts.

**Conclusion:**

We found patterned differences in how the participants described *leadership* and *decision-making* that may be related to participants’ professional training/background. As it can create misunderstandings and unsafe situations if nurses and physicians disagree on the meaning of *leadership* and *decision-making* (without necessarily recognising this difference), it could be beneficial to educate healthcare professionals to be aware of the specificity of their own concepts, and to communicate what exactly they mean by using a particular concept, e.g. “I want you to coordinate tasks” instead of “I want better leadership”.

**Supplementary Information:**

The online version contains supplementary material available at 10.1186/s12909-024-05682-x.

## Background

Social and cognitive capabilities are essential for safe and proficient patient care and treatment [1–3]. Traditionally, these capabilities have been called ‘non-technical skills’, but concern has been raised that the term is inadequate as it downgrades the value of the capabilities and defines them by what they are not instead of what they are [4]. Therefore, we have adopted the terminology *social and cognitive capabilities*. Social and cognitive capabilities include the ability to lead, communicate, make decisions, form an understanding of the situation, or work together in a team [5–11]. The social and cognitive capabilities have earlier been described in an array of models specific to certain medical fields (under the label of non-technical skills models or NTS model) [6, 8–11]. Each model contains four categories, several elements under each category, and behavioural markers for each element, which together explain vital social and cognitive capabilities within that field. The models have been used for teaching and assessment purposes [12, 13].

To the extent that social and cognitive capabilities can be analytically separated from so-called technical skills, studies have shown that issues related to social and cognitive capabilities contribute to up to 2/3 of errors and adverse incidents in hospitals [14]. Early work on this was done at the end of the 1970s and was intensified with the landmark report “To Err is Human” by the Institute of Medicine in the USA [15, 16]. Despite efforts to improve safety, unsafe events in hospitals are still a significant problem [17, 18].

Many factors could contribute to an ongoing issue regarding the role of social and cognitive capabilities in patient safety. Some studies seem to indicate that different groups of healthcare professionals might understand and apply certain social and cognitive capabilities – like teamwork, decision-making, and leadership – differently [19, 20]. If different groups of healthcare professionals act differently in relation to social and cognitive capabilities, such as leadership and decision-making, it could mean that their concepts behind the capabilities differ. Souba [21] argued that how we understand and think about a certain word, for example *leadership*, will influence how we act, how we speak, and what attitudes we have. Following that reasoning, we argue that healthcare professionals’ internal understanding of the terms behind social and cognitive capabilities could be coupled with the way they enact particular social and cognitive capabilities. Such internal understandings of words or terms might be referred to as concepts [22, 23], mental models [24], prototypes [25], and schemata [26]. Here, we call such internal understandings *concepts*. Every experience will potentially work to adjust an individual’s concepts. Some experiences can be designed specifically to form or adjust concepts. This is the case for experiences gained during education. In healthcare education, the concepts of *leadership* and *decision-making* will come up in many courses. The overt curriculum in these courses will obviously work to form and adjust concepts, but the hidden curriculum of the courses will also be influential [27, 28].

The hidden curriculum is a term used to describe cultural and social norms taught implicitly to students through experiences [27, 29]. This can either be their experiences in clinical practice or their experiences in teaching situations, such as how the educator uses and describes concepts. Some research suggests that the hidden curriculum is a more powerful determinant of later behaviour than the formal curriculum [28, 30]. Any education situation that health care professionals meet during their lifelong education will have an overt curriculum. This could be a plan of activities to teach them something about leadership. The experience will however also have a hidden curriculum, such as the valence ascribed to different leadership styles based on the educator’s personal preferences [27, 31]. Since education, including both formal and hidden curricula, work to shape concepts, educators play a key role for concept formation among healthcare professionals.

Based on Souba’s [21] observation that our understanding of a concept will influence how we act, we wish to examine variations in how educators at Copenhagen Academy for Medical Education and Simulation (CAMES) talk about social and cognitive capabilities. These potential differences are of interest as they will form part of a hidden curriculum within the courses taught by the educators [27, 31]. As such, variations will potentially result in different learning outcomes for the learners, which in this case means different understandings of the concepts behind social and cognitive capabilities. Different understandings of the concepts will mean different actions [21, 32], which could be part of the explanation for the differences we see in clinical practice as well as the cause of misunderstandings and unsafe situations. It is within this line of thought that we investigate differences in the ways that nurses and physicians speak of leadership and decision-making.

## Method

This qualitative interview study was carried out from February to August 2019. The aim of the study was to examine variations in how educators at CAMES talk about social and cognitive capabilities. The study was conducted using semi-structured interviews [33] and directed content analysis [34]. Codes for the analysis process were derived from existing NTS models and applied to show variations in how the participants talk about the same concepts. The Regional Ethics Committee of the Capital Region of Denmark waived the ethical review of the study (H-19,023,177). Participants received written and oral information about the purpose of the study, and all participants signed a written consent form before the interview took place. Data is reported using the COREQ checklist [35].

### Research team and reflexivity

FZ, an organizational psychologist, conducted the interviews. At the time of the interviews, FZ was a relatively new colleague of the interviewees and new to the healthcare system, which allowed him to be unbiased by previous healthcare experiences when interviewing and asking clarifying questions. FZ worked alongside the participants but was not directly involved in their work. FZ has conducted interviews in both clinical and work and organisational settings. RH and BB coded the interviews, and RH conducted the preliminary analysis. RH is a cognitive psychologist who has only recently started working within the healthcare system. This was utilised as a strength, as it made it easier for her to notice and be curious about tacit information and patterns in the interviews during the coding and the analysis. RH has worked with tacit information on earlier projects within other fields. BB is an anthropologist (PhD) with qualitative research experience within the healthcare system. Her experience and attention to the situatedness of discourse sharpened the analysis and placed it within current debate in health professions education research. PD is a work and organisational psychologist (PhD) who has worked within the healthcare system for about 22 years. His extensive knowledge was used to place our findings in a theoretical and practical frame within the field. The mix of researchers new to the field and the experience contributed by others, allowed us not only to see new perspectives, but also to situate them within current debates about social and cognitive capabilities [4].

### Study participants

The study is based on interviews with healthcare educators from CAMES, an influential health professional education institution in Denmark and internationally. CAMES has approximately 10,000 course participants per year, about 110 facilitators, and about 20 course directors. In addition, almost 150 simulation facilitators are trained per year in train-the-trainer courses at CAMES. Taken together, educators at CAMES have the potential to affect conceptualizations in many healthcare professionals in Denmark and thereby potentially influence their work in the Danish healthcare system.

The study participants are 11 course directors at CAMES. Course directors at CAMES organise courses and are responsible for content, programme, materials, and externally recruited educators related to the courses. The course directors involved in this study were approached based on their engagement in courses teaching social and cognitive capabilities. We decided to interview course directors, since their articulation of social and cognitive capabilities will likely influence how those capabilities are taught in their courses (e.g., through selection of educators, content, and materials). In this way, course directors are placed in a position where their knowledge and articulation potentially influence course participants’ learning and subsequent clinical practice. The participating course directors had at least one year of experience with teaching cognitive and social capabilities in simulation-based settings. All of them had a clinical background as nurses (six) or physicians (five). Ten course directors were women. All invited participants agreed to take part in the study and did so voluntarily. No one dropped out of the study.

### Data collection

Data was produced through semi-structured interviews [33]. We chose this interview method since we were interested in studying how course directors describe social and cognitive capabilities without their descriptions being influenced by a specific teaching situation, where many social factors might influence how a certain concept is spoken about. We asked course directors to talk about their teaching practice and the concepts they teach in their courses. With this prompting their descriptions of the concepts would likely reflect that context. The study was presented as part of ongoing efforts to develop teaching quality at CAMES.

Each interview focused on investigating the course director’s articulation of the central categories in a model of social and cognitive capabilities (NTS model) of their own choice (see interview guide in Appendix 1). We interviewed the course directors based on the following models: ANTSdk, N-ANTS, and NOTSSdk [8, 10, 36]. The categories and elements (marked by bullets) in each model are shown in Table 1. Each interviewee was asked open-ended questions to describe each category in the chosen model, and the categories were later used as initial codes for the directed content analysis inspired by the analysis process as described by Hsieh and Shannon [34].

**Table 1:** The three NTS models used in the interviews [8, 10, 36]

**ANTSdk**	**Situation awareness** • Gathering information • Recognising and understanding information • Predicting and thinking ahead • Exhibit self-insight	**Decision-making** • Identifying options • Choosing, communicating, and implementing decisions • Re-evaluate decisions	**Teamwork** • Exchanging information • Coordinating activities • Assessing capabilities • Supporting others	**Leadership** • Planning and preparing • Prioritising • Identifying and utilising resources • Using authority and assertiveness • Setting and maintaining standards
***N*** **-ANTS**	**Situation awareness** • Gathering information • Recognising and understanding information • Anticipating and thinking ahead	**Decision-making** • Identifying options • Assessing and weighing up options • Reassessing decisions	**Teamwork** • Exchanging information • Assessing roles and competencies • Coordinating activities • Displaying authority and strength • Exhibiting team behaviour and support for team members	**Task management** • Planning • Setting priorities • Making use of resources • Maintaining standards
**NOTSSdk**	**Situation awareness** • Gathering information • Understanding information • Predicting and thinking ahead • Monitoring own performance	**Decision-making** • Considering options • Selecting and communicating decisions • Implementing and assessing decisions	**Teamwork & communication** • Exchanging information • Establishing a shared understanding • Coordinating activities	**Leadership** • Setting and maintaining standards • Supporting others • Coping with pressure

The categories of each NTS model are shown in bold, and the elements under each category is marked by bullets

The interviews were carried out at the participants’ workplace, CAMES Herlev Hospital, in an interview room separate from the participants’ workstations and colleagues. In all interviews, only the participant and the interviewer were present in the room. The interview guide was formulated and validated by the authors as well as pilot tested with the first interview. Each interview, lasting between 20 and 30 min., was recorded and afterwards transcribed verbatim. The interviewer took notes during each interview. Interviews, notes, and transcriptions were produced in Danish. Only extracts presented in this article were translated into English. Once the transcriptions were completed, they were returned to the participants for the participants’ review to suggest corrections or comments. As no comments or corrections were provided on the transcriptions, and since authors agreed that data saturation had been reached, it was decided not to carry out any repeat interviews.

### Data analysis

Data was analysed using directed content analysis [34]. We chose this method because we wished to extend our current understanding of well-used concepts [34]. We used the NTS models as a framework (Table 1). Analysis was carried out using the NVivo software (www.lumivero.com) based on the following steps: analysis prior to and during data production, workshop with interviewees, coding of transcripts, looking at patterns, recoding of N-ANTS interviews, and consensus.

#### Step 1: analysis prior to and during data production

This being a qualitative study, data analysis started already with our choice of theoretical framework (content analysis), which directed our method (semi-structured interview) and the categories used in the interviews. The data production process has been further described in the section above.

#### Step 2: workshop with interviewees

After data production, the initial impressions from the interviews were discussed with the interviewees in a workshop led by FZ. The purpose of the discussion was to bring to light new or missed perspectives from the participants. The workshop was valuable for the participants, but it did not uncover new perspectives for the analysis.

#### Step 3: coding of transcripts

**Fig. 1:**
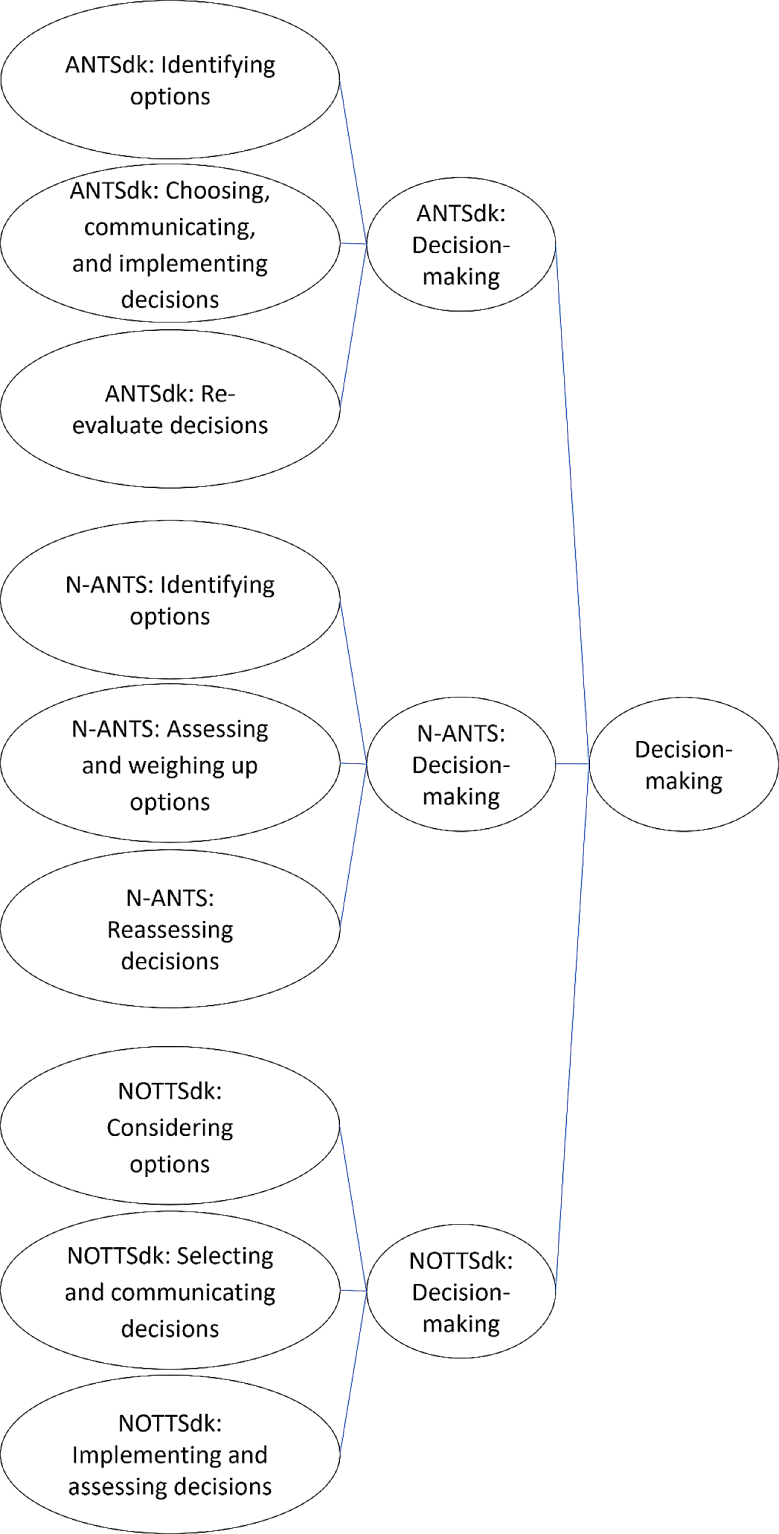
Coding tree for *decision-making*

Inspired by the analysis process described by Hsieh and Shannon [34], RH and BB coded the transcriptions using the elements from the NTS model on which the specific interview was based as initial codes. A coding unit could be one or more sentences or pieces of sentences. A coding unit was coded if it used the specific words of an element from the NTS model used, or if the meaning was judged to be fitting with the meaning of the element. Coding units could be coded to fit into more than one element. In some cases, an interviewee talked about a category from the chosen model without it belonging to a clear element within that model. In these cases, the unit was coded as the category from the model instead of a concrete element. Furthermore, we coded each coding unit based on whether the interviewee was talking about the individual, the team, the organisation, or society. RH and BB discussed differences in their coding, and a shared understanding was reached in each case. Immediately after the first round of coding, we coded all interviews again to check the consistency of the coding. An example of a coding tree for *decision-making* is shown in Fig. 1. Similar coding trees were used for the four other categories and their elements.

#### Step 4: looking at patterns and step 5: recoding of N-ANTS interviews

During the analysis, we looked at different constellations in the dataset to find patterns in the variations of how the participants talked about the concepts. In this process, we became interested in *leadership* and *decision-making* as we found a clear pattern in relation to these categories. This pattern made us to look at the dataset again, as *leadership* is not a category within the N-ANTS model. For this reason, we looked through the N-ANTS interviews again and found all the paragraphs concerning leadership. We included these paragraphs in our “*leadership* data”. We decided not to continue working with *situation awareness*, *teamwork*, and *task management* as these categories did not show the same pattern as found in *leadership* and *decision-making*. Even though we found the pattern only in two of the categories, we argue that the finding is sufficient to establish the possibility that differences in understanding of words exist in the healthcare system.

#### Step 6: consensus

Throughout the analysis, we examined the transcripts repeatedly to find examples to substantiate or reject the observed patterns. This process involved all authors. We discussed findings and agreed that our data could substantiate the observed patterns sufficiently. We presented our preliminary findings and analysis to the group of participants in the study. Participants’ feedback was acknowledgement and recognition of the observations presented.

## Results

In this study, we carried out 11 semi-structured interviews with course directors involved in teaching and planning education related to social and cognitive capabilities. Using a directed content analysis [34], we examined patterns in variations in how participants talked about concepts in three NTS models; ANTSdk, N-ANTS, and NOTTSdk [8, 10, 36]. During this process, we noted how course directors with professional backgrounds as nurses and course directors with professional backgrounds as physicians described the two categories *decision-making* and *leadership* both similarly and differently. Similarities included a pronounced interweaving of course directors’ concepts and definitions of the concepts as per the NTS models. Differences between nurses and physicians included variations in understanding of role distribution (e.g., who could be the leader), focus on external versus internal factors, and the ascribed importance of group dynamics versus individual capabilities. Course directors with a background as nurses generally thought that nurses could be good leaders, and they generally paid greater attention to group dynamics, hierarchies, and external factors. Course directors with a background as physicians tended to think that leaders needed to be physicians, and they focused on their individual efforts when they talked about *decision-making* and *leadership*. In the following sections, we elaborate on these findings by first showing how the course directors – nurses and physicians alike – describe *decision-making* and *leadership*. Afterwards, we elaborate on patterns in how nurses and physicians describe the two categories differently.

### Leadership & decision-making among healthcare professionals

Both nurses and physicians linked *leadership* and *decision-making*, and they made connections between these two categories. When asked to describe *leadership*, there were interviewees, who started talking about a leader (typically the team leader) as a person, whereas other interviewees talked about leadership as an activity. Only one interviewee talked about followership when describing *leadership*. A team leader was described as someone who collects information about what is going on and maintains an overview of the situation:

“*I perceive the team leader position as having to keep an overview, maintaining the overview, you have to keep track of your team members. You have to keep your hands in your pockets, so that you can maintain an overview and not get caught in a heads-down procedure*” (physician 1).

Furthermore, a team leader was described as responsible for creating a calm, comfortable, and safe atmosphere within the team: “*It is important that you create a pleasant and calm atmosphere around the patient, and that you give your team members a good feeling, so that they won´t become stressed, but can function optimally*” (physician 1). Both nurses and physicians stressed that the team leader should be the person most competent to do the job, and that it can be a problem if someone is appointed leader without the necessary qualifications:

“*Yes, I think it takes professional skills to be the leader in an emergency. It can probably be discussed whether leadership only requires an overview - what gives you that overview? That is a matter of having competencies within the situation, and for that reason it is traditionally the most competent person who gets the leader role. It can become a problem, if you get the leader role based on your profession and not on your competencie*s” (nurse 4).

Interviewees from both professions talked about *leadership* entailing authority. All participants mentioned every element of *leadership* from their respective NTS models, but it is interesting to note that three nurses were interviewed based on N-ANTS, which does not contain *leadership* as a category. Still, these nurses referred to *leadership*: “*So it is both about prioritising what we need to do first and what we need to do afterwards, but also about thinking of delegating: what can I do myself, and what do I want you to do*” (nurse 3).

*Decision-making* was described as requiring extensive experience and a strong theoretical background – either by the decisionmaker him- or herself, or that the decisionmaker must include knowledge from experts/the team in the decision-making: “*If you have seen 5000 patients being anesthetised in the same way, then you have seen some things and some patterns, and that makes it possible for you to make the right decision*” (nurse 5).

“*As the team leader, I have other tasks. [For example: ] I don’t know which antibiotic to give, and now I have to spend time to look it up – I don’t want to do that. I have a competent person who can do it by virtue of his/her knowledge, so I delegate that task, and then they also have to make the decision about it*” (physician 1).

For this reason, inexperience was mentioned as a potential problem for decision-making. Both nurses and physicians mentioned that inexperienced decisionmakers could have problems recognising the relevant patterns to adequately understand the situation and make the right decisions:

“*When you are a novice and new in a profession, sometimes it is difficult to know what situation you are in, and what decision you should take, because you don’t have the necessary knowledge, or you haven’t seen enough examples of what other possibilities you have*” (nurse 5).

“*It might happen that you don’t dare to make a decision, or that you are not capable of it, because you get so perplexed by all the incoming information when you are standing there with your situational awareness, and you get all this information from your team and how… you cannot recognise patterns, for example*” (physician 3).

Another challenge that might hinder decision-making, according to the interviewees, was the social hierarchy. An example given by the interviewees was how knowledge in the team can be lost if some members of the team (e.g., nurses or junior physicians) are not heard because of social hierarchy, or if they do not feel comfortable sharing their opinions or observations. Lost knowledge potentially leads to poorer decision-making.

“*There can be some hierarchy in it, just because physicians are worth more than nurses, for example. There can also be some hierarchy in whether you are new or experienced. And then there can be a learning culture in a ward, which can result in it not being very welcome to ask all those questions*” (nurse 6).

Interviewees warned against fixation errors in relation to decision-making. They talked about the risk of being wrong when making a decision, and about how it is important to constantly keep an eye on different possibilities: “*We see some systems which fit into the puzzle, and then we think it is like that, and then we go that way and don’t see that the puzzle could be laid in another way*” (physician 4).

*Decision-making* was, according to the interviewees, related to both *teamwork*, *leadership* and *situation awareness*, and all interviewees mentioned every element under the category *decision-making* in their respective non-technical skills models (see Table 1).

### Differences between nurses and physicians

While working with data, we became aware of patterns in the way that nurses and physicians talked differently about the categories in the context of their work life. These differences were not based solely on the content of specific categories, but on differences in what nurses and physicians focused on and showed interest in.

Nurses talked about *leadership* and *decision-making* as something every team member takes part in, and as something every team member is responsible for. Nurses described *decision-making* as something the entire team contributes to: *“(…) but I find decision-making isn’t necessarily placed only with the leader, it is placed with every team member in which direction you go*” (nurse 2). Similarly, nurses talked about *leadership* as something both nurses and physicians enact. Sometimes it was described as different kinds of leadership: “*Because the scrub nurse can really have a lot of leadership in the operating room. That is, the inventory and the accessories and what goes in and out. And who should be called to assist and when. And there is a great deal of leadership in that*” (nurse 2).

Another nurse talked about how nurses can sometimes be the best team leader, for example in a staff constellation of a senior nurse and a junior physician. By contrast, physicians described *leadership* and *decision-making* as something the physician does (alone). Their focus was on the role of the physician and the physician’s responsibilities:

“*It is also how the team leader attains a position of authority, as he/she should have, which is especially difficult in a paediatric ward, unlike perhaps… what do I know, surgical wards, it doesn’t feel natural for paediatricians to have authority and assertiveness, because our daily tone is very non-hierarchical and with very little authority and assertiveness from physicians, from where the team leader must be recruited*” (physician 5).

Interestingly, a physician referred to the same staff constellation as described by a nurse, i.e., a situation where a senior nurse and a junior physician would be working together, but the physician described how it is important to teach the junior physician to lead and the senior nurse to respect the leadership.

The tendency for nurses to be oriented towards the team and for physicians to be focussed on individual factors associated with the physician role was apparent throughout the dataset and across the different concepts. Both nurses and physicians talked about both individual factors and team factors, but the tendency was for nurses to talk more about team factors than individual factors, whereas the opposite tendency was found for physicians. An example of a statement related to the team would be: “*You need to include people from your team. Because they can have other information, they can have examined something else, they can have seen something else, heard something else*” (nurse 1).

Furthermore, only nurses talked about organisational factors and societal factors: “*There can be something organisational in task management. We don’t have the resources that we need, we don’t have the equipment that we need*” (nurse 1).

“*When we fixate on something, what we call fixation errors, (…) we actually produce it ourselves in our system in the way patients enter our hospitals. (…) So, we need to work on these concepts [social and cognitive capabilities], because our system is taking part in producing them [fixation errors], like we ourselves can take part in producing them [fixation errors]*” (nurse 6).

Contrary to the nurses’ broad focus on external factors, physicians talked more about the individual physician and internal factors, such as personal growth, how to advance from inexperienced to experienced, individual responsibility, and how to step up and be the team leader, etc.:

“*I think, as a specialist, if they master these [NTS] concepts early, then they get the space to develop the role and to set themselves in the process. This is where the problem sits, I think. It is when the individual and the role get mixed. That is also when it becomes unsafe for patients*” (physician 2).

When talking about *leadership* and *decision*-*making*, we observed a tendency in the data towards nurses talking more about social hierarchies in hospitals than physicians did. When social hierarchies were mentioned, it was mostly with a negative valuation. Social hierarchies were described in relation to seniority, where the senior (experienced) individual would be higher up the hierarchy than the junior (inexperienced) individual, and in relation to nurses and physicians, where physicians would be higher up the hierarchy. Both physicians and nurses talked about the problem of working with someone high in the hierarchy if that individual did not have the required competencies to fill that position:

“*Sometimes there is a formal leader who does not have the necessary qualifications. It is often the problem that there is someone who formally in the hierarchy of the hospital should be team leader, but in reality they are not ready for it at all, and other team members would be able to manage that task better – that is a problem*” (physician 1).

There were nurses who talked about a flatter hierarchical structure, where nurses or young physicians could be team leader, and even structures without any leader at all. One nurse described how the hierarchical structure in the hospital was becoming flatter as a result of a general development in society:

“*I think this hierarchy is evolving. In the past, the chief physician was someone who just stood with folded arms and the nurses ran around. That’s not the case anymore. It has increasingly become a collaboration where you get an understanding that you need each other. I think the whole development in society means that you flatten some of the hierarchy that has existed in the past*” (nurse 6).

## Discussion

This study used semi-structured interviews [33] and directed content analysis [34] with the aim to investigate patterns in variations in how healthcare educators talk about *leadership* and *decision-making*. The main findings from the study show how educators with backgrounds as nurses and physicians respectively talked differently about *leadership* and *decision-making*. The nurses in the current study described both *leadership* and *decision-making* as something the whole team engages in, whereas the physicians talked about them as something the physician does (alone). The nurses thought that nurses could be the team leaders, whereas physicians mentioned that the team leader must be a physician. The nurses talked more about group factors than individual factors, and they mentioned both organisational and societal factors. The physicians talked more about individual factors than group factors, and they did not mention organisational or societal factors. The nurses talked more about social hierarchies than the physicians did, and the hierarchies were almost always talked about as negative. The study contributes to the existing literature by showing that there are patterned differences in the way educators with a background as nurses and physicians respectively talk about *decision-making* and *leadership*. We argue that these differences might be passed on to the students through teachings. The main findings resonate well with previous studies on behavioural differences between nurses and physicians. This strengthens our argument that differences in understanding of concepts might underlie differences in behaviour which might again lead to safety issues. Thus, safety issues might be compounded by educators’ different understandings of concepts. We will discuss this further below.

Previous studies have found differences between nurses and physicians that are in line with our findings. Barrow and colleagues [19] found that nurses in their study thought they enacted leadership and decision-making, whereas many of the physicians in the study directly disagreed with that. Likewise, the majority of physicians in the study thought that the effectiveness of interprofessional teams relied on strong leadership from physicians [19]. Our findings suggest that such disagreement in clinical practice might be rooted in differences in how each profession understands the concepts they disagree about. On the other hand, research has also shown that while physicians exercise “direct” decision-making, nurses apply covert strategies like selecting information given to the physicians to try and steer the physician in the direction of the “right decision” [37, 38]. By using a covert strategy for decision-making, it is probable that physicians do not even realise that the nurses are making decisions (or have a part in the decisions taken), which could also be (part of) the reason why nurses and physicians think differently about who makes decisions. Similarly, Barrow and colleagues [19] described different decision-making and leadership strategies for nurses and physicians, with nurses using external factors as their powerbase for authority and leadership. For example, nurses could say that something should not be done due to current guidelines, or they could approach another physician after shift change if they disagree with a decision (this observation is backed up by Svensson [39]). These differences in enactment of leadership and decision-making could grow from variations in understanding of the concepts. For example, nurses could be oriented toward external factors in their understanding of the concepts and physicians toward internal factors, as our study finds.

Extending the differences between external and internal factors, one of our main findings show variations in how much nurses and physicians talk about group factors and individual factors. While we have not found any other research analysing the tendency to talk about individual versus group factors among nurses and physicians, some studies have shown that physicians focus on each individual in the team instead of the group as a whole when describing ‘team’ and ‘teamwork’ [40]. Another study has shown that physicians talked about leadership as a group process when they were asked to define leadership, but as a personality trait when they simply talked unsolicited about leadership [41]. These findings could be a result of a physician tendency to focus on individual factors when defining concepts related to leadership.

Our findings further indicate variations in understanding of and interest in hierarchy. Several earlier studies showed an effect of hierarchy on how healthcare professionals understand and exercise social and cognitive capabilities [20, 42]. Makary and colleagues [20] found that physicians and nurses in the operating room evaluated their teamwork differently with physicians rating it higher than nurses. Some of the explanations suggested by the authors involved how social status and hierarchies might influence how healthcare professionals perceive teamwork, but also that physicians and nurses might have different ideas about what constitutes good teamwork [20]. The latter would be in line with our argument in this study. However, hierarchy might also be another explanation as to why differences in behaviour appear in clinical practice. Research has shown instances where hierarchy can influence decision-making by showing how nurses are constrained by physicians in their decision-making, but not the other way around [43].

Our study indicates that nurses and physicians understand *leadership* and *decision-making* differently, which resonates well with earlier studies. Our participants worked as healthcare educators involved with teaching and planning courses concerning leadership and decision-making among other topics. Previous research has found that teachers’ preconceptions, preferences, and biases can form a hidden curriculum within a course [27, 29, 31], and that a hidden curriculum can be a powerful determinant of later behaviour [28, 30]. Seeing our findings in this light, we argue that it is likely that the differences in understanding of *leadership* and *decision-making* among the educators in our study will form a hidden curriculum in their courses. They might choose certain cases for a simulation session or focus on a specific event in the debriefing, which will advance their particular understanding of a concept. Or they might use different words or emphasis when explaining a concept. Such a hidden curriculum can influence learning and later behaviour among course participants. An example of this was shown in a study by Ju and van Schaik [25] on leadership prototype formation (the understanding of what it means to be a leader). Ju and van Schaik [25] argued that prototype formation is influenced by the teaching materials and role models that health professionals are exposed to during their education and clinical practice. Even something as simple as the sex of nurses and physicians in educational videos could have an impact on prototype formation and later behaviour [25]. We argue that the observed differences in understanding of *leadership* and *decision-making* would similarly influence course participants’ concept formation (or ‘prototype formation’ to use the terminology of Ju & van Schaik [25]), which would cause course participants taught by a nurse educator to form a slightly different understanding of, for example, *leadership* than a course participant taught by a physician educator. These differences would later cause the course participants to act differently in clinical practice [21, 32, 44], which could potentially lead to miscommunication and misunderstandings between nurses and physicians. Nurses and physicians work together every day in clinical practice, and even minor disagreements about who decides what, and who leads whom can lead to frustration and unsafe situations [19]. If nurses and physicians do not agree on their respective roles, leadership could for example become unclear in an emergency situation where too many or too few step up to the task [45]. Alternatively, physicians might make a decision, since they think it is their responsibility, and nurses, who are left out of the original decision-making process, might undermine it or work against it based on frustrations resulting from differences in concepts (as seen in [19]). Since much nurse and physician learning happens in clinical practice through experience and observation of others [46], differences in behaviour would reproduce differences in understanding of the concepts. This is particularly pertinent for “newcomers” learning the language and legitimate actions of a workplace [47]. Novice nurses see how other nurses talk and act in clinical practice and then adapt their language and behaviour based on these interactions to fit into the observed community [47]. In this way, certain understandings of *leadership* and *decision-making* would be reproduced.

Examples of potential miscommunications are already evident in the present study. A withdrawn leader is both described positively (a good leader position with overview) and negatively (as someone just bossing the team members around, without engaging in helping the team). Becoming aware of these different understandings is a first step towards a deeper understanding and better communication among different groups of healthcare professionals, which could potentially alleviate conflicts and improve patient safety.

### Limitations

It is important to mention that the differences we have observed between nurses and physicians in our study might have originated from other characteristics of the participants than their professional background. Examples could be their level of experience as clinicians or as educators, the nature of the courses they teach, the participants in their courses, gender (though unlikely, as the study participants included only one male), or personality traits.

Note that we asked participants to talk about the concepts in the context of their teachings, which might be different from how they would talk about the concepts in another setting, or how they would use the concepts in clinical practice. It would have been beneficial to supplement our interview data with observations of teaching practices or clinical work.

Furthermore, we base our considerations on a small data set, but considering the match between our findings and aspects described in the literature, we see support for our findings and interpretations.

## Conclusion

In this study, we found that nurse and physician healthcare educators to a large extent described social and cognitive capabilities as they are described in existing tools addressing non-technical skills. We also found patterned differences in their descriptions that may be related to educators’ professional training/background. Focusing on the concepts *leadership* and *decision-making*, nurses paid greater attention to group dynamics and external factors, whereas physicians focused on their individual efforts. If nurses and physicians disagree on the meaning of leadership and decision-making, for example regarding who should decide in a given situation, it can create misunderstandings and unsafe situations. For this reason, it could be beneficial to make healthcare professionals aware of the specificity of their own concepts, so that they can communicate better about meanings and differences of concepts in teamwork situations. This could be done by educating them to describe more precisely what they mean when using a certain concept, for example “I want you to coordinate tasks” instead of “I want better leadership”. In this way, we might avoid healthcare professionals using the same word, but in fact referring to different concepts.

### Electronic supplementary material

Below is the link to the electronic supplementary material.

Supplementary Material 1

## Data Availability

The data used in this study are not publicly available due to the sensitivity of the data. It will not be possible to obtain the raw data upon request, as neither the participants nor the institution have agreed to have the raw data shared. Questions regarding the data or requests to see the data can be addressed to the corresponding author.
