# Supplementary material for: An interview study about how nurses and physicians talk about the same concepts differently
Source: BMC Med Educ. 2024 Jun 26;24:698. doi: 10.1186/s12909-024-05682-x (PMC11210097; doi:10.1186/s12909-024-05682-x)
Supplement: Supplementary file 1 — Supplementary Material 1 [file 12909_2024_5682_MOESM1_ESM.docx]

# Appendix 1 - Interview guide

**Introduction to interview**

“*At this interview I am going to ask you to describe your understanding of central notions within Non-Technical Skills and Human Factors. It is not an exam, and there are no right or wrong answers*”.

**What model do you work with?** (Show the concrete models on a piece of paper)

**What do you understand by the words in ANTS etc.?**

**What do you understand by Teamwork?**

**What is good and bad performance within this category?**

**What does Teamwork mean for you?**

**What do you understand by Decision Making?**

**What is good and bad performance within this category?**

**What does Decision Making mean for you?**

**What do you understand by Leadership/Task Management?**

**What is good and bad performance within this category?**

**What does Leadership/Task Management mean for you?**

**What do you understand by Situation Awareness?**

**What is good and bad performance within this category?**

**What does Situation Awareness mean for you?**

**If you had to choose, which of the categories in the Non-Technical Skills model would you judge to be most important? If you had to choose, how would you prioritise the categories and why?**
